# Sex Differences in Pulmonary Hypertension

**DOI:** 10.3389/fragi.2021.727558

**Published:** 2021-10-04

**Authors:** Juan José Rodriguez-Arias, Ana García-Álvarez

**Affiliations:** ^1^ Cardiology Department, Institut Clínic Cardiovascular, Hospital Clínic, IDIBAPS, Madrid, Spain; ^2^ Universidad de Barcelona, Barcelona, Spain; ^3^ Centro Nacional de Investigaciones Cardiovasculares Carlos III (CNIC), Madrid, Spain; ^4^ Centro de Investigación Biomédica en Red (CIBER) de Enfermedades Cardiovasculares, Madrid, Spain

**Keywords:** estrogens, pulmonary hypertension, estrogen paradox, animal models, sex, estrogen receptors, pulmonary hypertension groups

## Abstract

Pulmonary hypertension (PH) includes multiple diseases that share as common characteristic an elevated pulmonary artery pressure and right ventricular involvement. Sex differences are observed in practically all causes of PH. The most studied type is pulmonary arterial hypertension (PAH) which presents a gender bias regarding its prevalence, prognosis, and response to treatment. Although this disease is more frequent in women, once affected they present a better prognosis compared to men. Even if estrogens seem to be the key to understand these differences, animal models have shown contradictory results leading to the birth of the estrogen paradox. In this review we will summarize the evidence regarding sex differences in experimental animal models and, very specially, in patients suffering from PAH or PH from other etiologies.

## Introduction

Sex constitutes a non-modifiable risk marker for several diseases (Khamis et al., 2016; Mattiuzzi and Lippi, 2019) but in few of them its consequences represent such a challenge as in pulmonary hypertension (PH). Differences related to sex in PH are not only present in its prevalence but also in its severity, response to treatment and survival.

PH defines a heterogeneous group of diseases characterized by elevated pulmonary artery pressure (PAP). Current guidelines and the recent 6th World Symposium on PH defines five groups of this entity based on differences in pathological findings, hemodynamic characteristics, clinical presentation and treatment approach (Simonneau et al., 2019).

Complex mechanisms involving a dysregulation of nitric oxide, endothelin-1 production and the intervention of multiple cell types like pulmonary artery smooth muscle cells (PASMCs), pulmonary artery endothelial cells (PAEs) and fibroblasts constitute the hallmarks of pulmonary arterial hypertension (PAH), although they are involved to a lesser or greater degree in all cases of PH (Humbert et al., 2019). Other specific changes are observed depending on the etiology. Thereby, in the case of PH due to left heart disease, a backward pressure transmission from the left side of the heart is the main pathological mechanism, which is associated with chronic changes in the pulmonary vasculature in a substantial percentage of patients who develop combined pre and postcapillary PH (Vachiéry et al., 2013). Conversely, in PH associated with lung disease a muscularization of pulmonary arteries and arterioles due to chronic hypoxia and inflammation seems to be the main cause (Smith et al., 1992). On the other hand, chronic thromboembolic pulmonary hypertension (CTEPH) is typically associated with incomplete resolution of prior venous thromboembolism episodes. This chronic obstruction leads to a histological adaptation of the microvascular arterioles and a progressive rise in pulmonary vascular resistance (PVR) (Delcroix et al., 2016). Globally, in the long-term, remodeling of the lung vasculature is the main cause of a rise in PAP and final right ventricle (RV) disfunction. In the first stages of the disease, the RV adapts to the increased afterload developing myocardial hypertrophy and augmented contractility due to changes in myosin isoform expression (Ryan and Archer, 2014). Despite these changes, if afterload is not reduced, a maladaptive adaptation of the RV leads to its failure (Ryan and Archer, 2014). RV disfunction constitutes a major determinant of survival in PH (Jacobs et al., 2014).

Incidence and prevalence are clearly dependent on the group of PH, from 6 to 25 cases per million in the case of PAH (Badesch et al., 2010; Ling et al., 2012) to a prevalence as high as 70% of all patients with heart failure (HF) in the case of PH secondary to left heart disease (Miller et al., 2013; Shah et al., 2014). However, a bias towards a higher prevalence in women is clear, especially in PAH (Badesch et al., 2010; Hoeper et al., 2013).

In this review we aim to recapitulate the evidence regarding the complex relationship between hormones and sex bias in both animal models and patients suffering PAH, or other causes of PH.

## Overview of Sex Hormones Synthesis and Metabolism

**FIGURE 1:**
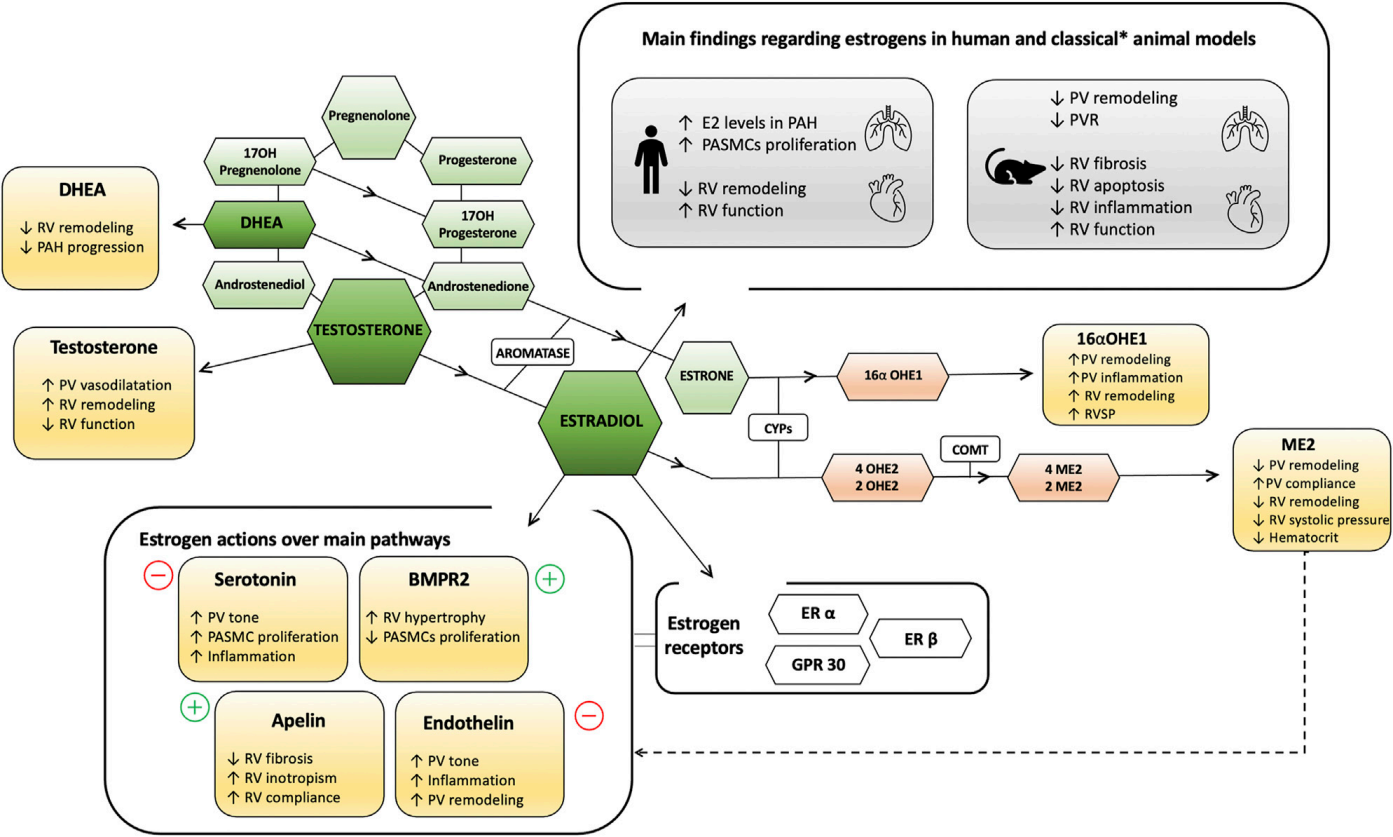
Overview of sex hormones synthesis, metabolism and main effects in humans and animal models. Rectangles refer to main functions. Coloured exagonal forms refer to hormones and metabolites, and non-coloured exagonal forms to enzymes. * Opposite results have been observed in the model of SERT and S100A4/Mts1. +/- referes to upregulation and downregulations of the molecule activity descrived, respectively. 2OHE2, 2 hidroxyestradiol; 2ME2, 2-methoxyestradiol; 4OHE2, 4 hidroxyestradiol; 4ME2, 4-methoxyestradiol; 16αOHE1, 16 α-hydroxyestrone; BMPR2, Bone Morphogenetic Protein Receptor Type 2; COMT, Catechol-O-methyltransferase; CYPs, cytochrome enzymes; DHEA, dehydroepiandrosterone; E2, estradiol; ER, estrogen receptor; GPR, G protein-couple receptor; OH, hydroxy; PAH, pulmonary arterial hypertension; PASMCs, pulmonary artery smooth muscle cells; PV, pulmonary vascular; RV, right ventricular.

A detailed description of sex hormone metabolism and signaling, published elsewhere (Austin et al., 2013; Lahm et al., 2014; Lahm and Kawut, 2017; Tofovic and Jackson, 2019; Tofovic and Jackson, 2020), is beyond the scope of this review. Briefly, the three main estrogenic steroids are 17-β estradiol (estradiol or E2), the primary active sex hormone in the female, estrone (E1) and estriol (E3). Cholesterol is the main precursor of these hormones after several enzymatic processes as the aromatization of the androstenedione and testosterone hormones (Nelson and Bulun, 2001). Dehydroepiandrosterone (DHEA) is a an intermediate hormone and precursor for both male and female hormones. Conversion of testosterone to E2 and of androstenedione to E1 is catalyzed by aromatase (CYP19A1). Figure 1 summarizes the complexity of sex hormones synthesis and metabolism as well as their main effects in humans and animal models. Other than ovaries, especially in men and postmenopausal women, estrogens production depends on peripheric tissues through the action of the aromatase enzyme (Nelson and Bulun, 2001). Thereby, E2 synthesis is possible in endothelia, vascular smooth muscle cells and myocytes (Harada et al., 1999; Simpson et al., 2002).

Estrogens levels vary among individuals, change with age, and in premenopausal women their concentration and type oscillate in a 28-days cyclical fashion (Draper et al., 2018). Estrogens exerts most of their biological effects through estrogen receptors (ER) ERα and ERβ, which are members of the nuclear receptor superfamily and are encoded by genes physically located in separate chromosomes (Heldring et al., 2007). In addition, a G protein-coupled receptor (GPR30) located in the cell membrane can also act as a binding receptor for estrogens (Pupo et al., 2016). In this setting two main signaling pathways are important, descripted in detail in previous dedicated reviews (Heldring et al., 2007; Murphy, 2012). A genomic pathway in which E2 dimerizes with ERα or ERβ and posteriorly acts as a transcription factor (Marino et al., 2006), and a non-genomic pathway through kinase activation and second messengers, such as activation of endothelial nitric oxide synthase (Lahm et al., 2008) or prostacyclin synthase (Frump et al., 2021) which effects take places in seconds or minutes (Shaul, 1999). ER are expressed not only in the reproductive system but also in the lung and cardiovascular tissues, in PAECs, PASMCs and fibroblasts (Dougherty et al., 2006; Hamidi et al., 2011). Due to this wide receptor expression, estrogens effects are not reserved to sexual processes and they are involved in the correct vascular and cardiac function as well as bone metabolism (Xing et al., 2009; Murphy, 2011; Streicher et al., 2017). Both, ERα and ERβ activation has been shown to attenuate injury-induced vascular remodeling through anti-inflammatory effects reducing the expression of TNFα (Xing et al., 2007), C reactive protein, and neutrophil chemotaxis (Miller et al., 2004); inhibition of neointima formation by inhibiting mitogenic effects of a number of growth factors such as FGF-2 on PASMCs (Suzuki et al., 1996); and modulating nitric oxide synthase (McNeill et al., 2002). However, the effects of ERs activation are cell specific and not completely understood. Moreover, their expression and activity are modified by multiple factors, such as sex, age, diet, variations in endogenous hormone levels (menstruation cycle, menopause or pregnancy) or disease states (Xing et al., 2009; Murphy, 2011). Indeed, opposite effects of E2 in aged versus young female animals have been found regarding inflammation and neointima formation (Miller et al., 2007; Suzuki et al., 2007; Laczy et al., 2009). A differential O-GlcNAcylation of critical proteins has been postulated (Laczy et al., 2009). Also DHEA levels declines with age (Kushnir et al., 2010). In addition to their effects on vascular remodeling and function, E2 has been described to have pro-contractile, anti-inflammatory and antiapoptotic effects in the RV (Frump et al., 2015; Lahm et al., 2016). Downstream mechanisms include ERα-mediated increased BMPR2 and apelin upregulation (known to be decreased in maladaptive RV remodeling) (Frump et al., 2021) and downregulation of proteins of the endothelin system (Nuedling et al., 2003).

Moreover, accumulating evidence indicates that the vascular effects of E2 are mediated largely by its downstream metabolites. Two seem particularly relevant in PH: 2-methoxyestradiol (2 ME), and the 16 α-hydroxyestrone (16αOHE1). 2ME is characterized by ER-independent antiproliferative, proapoptotic, anti-angiogenic and anti-inflammatory effects whereas 16αOHE1 promotes proliferative and proinflammatory processes (Dubey et al., 2004). In addition, CYP1B1 leads to the production of 4-hydroxyestradiol through oxidation of E2 at C4, which as reactive oxygen species carcinogenic effects. Growing body of evidence proposes that dysregulated E2 metabolism influences the development of PH and suggests a major pathogenic role for 16αOHE1 and CYP1B1, and 2 ME mediating some of the beneficial biological effects of E2 (Tofovic and Jackson, 2020). In this sense, CYP1B1 has been implicated in the increase in intracellular serotonin via increased serotonin receptor 5HT_1B_ (SERT) expression associated to PASMC proliferation (Johansen et al., 2016), and also in the metabolism of arachidonic acids into hydroxyeicosatetraenoic acids and epoxyeicosatrienoic acids, hereby stimulating PASMC growth, inflammation, hypoxic vasoconstriction and vascular remodeling (Jiang et al., 2011; Zhu and Ran, 2012). However, as detailed in depth in the review by Tofovic and Jackson (2020), there is no specific studies linking CYP1B1 and 16αOHE1 and there are still gaps of knowledge regarding the exact pathogenic role of both molecules in PAH in humans.

In the case of testosterone, its main production depends on the gonads and their levels decline with age (Araujo et al., 2004). Similar to estrogens, their function is not limited to sexual characteristics. Testosterone receptors are also expressed in the cardiovascular system and are related to the development of atherogenic plaques (Hanke et al., 2001) conferring a higher cardiovascular risk (Kloner et al., 2016).

## The Sex Paradox in Pulmonary Arterial Hypertension

PAH patient registries constantly demonstrate a female susceptibility with a higher female to male ratio, ranging from 1.4:1 in the UK/Ireland registry (10) to 4.1:1 in the REVEAL registry (11). The European COMPERA registry demonstrated a 1.6:1 female/male ratio globally however, a much greater difference was observed among younger patients (18–65 year-old) with a female to male ratio of 2.3:1 as compared to just 1.2:1 in those older than 65 (Hoeper et al., 2013). This equalization of female to male ratio is thought to have a relationship with changes in hormone levels related to menopause (Ventetuolo et al., 2014). On the other hand, registries demonstrate as well that female PAH patients consistently show better survival than men (Benza et al., 2010; Humbert et al., 2010; Olsson et al., 2014).

Sex differences in PAH have been expressed as a concept called the “estrogen paradox” summarized in two main points. First, even if a female susceptibility has been observed, once affected, women have a better response to treatment and survival than men. Secondly, animal models show both, deleterious and protective effects of estrogens over the lung vascular bed. The attempt to elucidate this concept has aroused great interest in the scientific community, leading to excellent reviews of the role of estrogens in experimental models of PH and in patients with PAH (Lahm et al., 2014; Docherty et al., 2018; Tofovic and Jackson, 2020). Given the rapid advance of knowledge, our objective is to review current evidence at an experimental and clinical level, as well as the results of clinical trials aiming to modify the estrogen axis carried out or in progress in PAH patients.

### Evidence From Experimental Animal Models of Pulmonary Arterial Hypertension

First animal models of PH were based on chronic hypoxia and monocrotaline (MCT) induced PH. Chronic hypoxia causes PH in rats and mice meanly through two mechanisms: erythrocytosis, leading to a higher blood viscosity (Vanderpool and Naeije, 2018), persistent hypoxic vasoconstriction and a variable degree muscularization of small arteries (Stenmark et al., 2009). In such model, females mice present a lower hematocrit level compared to male counterparts (Vanderpool and Naeije, 2018) and higher levels of pro-angiogenic factors at RV myocardium which might contribute to an improved RV adaptation (Bohuslavová et al., 2010). Similarly, female adult rats present better adaptation with lower PAP than males (Rabinovitch et al., 1981), that worsens after ovariectomy which may be attenuated through estrogens administration (Earley and Resta, 2002). In addition, very recently it has been shown that 2 ME abolishes hypoxia-induced PH in rats associated with a reduced protein expression of hypoxia-inducible factor 1α protein expression, a pro-proliferative mediator (Docherty et al., 2019), reversion of the downregulation of miR-223 (Hao et al., 2019), inhibition of endothelin gene expression (Earley and Resta, 2002) and reducing hematocrit (Tofovic et al., 2021), all suggesting that in female rodents female sex hormones are protective against PH. However, this model translates poorly to human PAH as it lacks some of the main human characteristics of PAH as plexiform lesions and severe RV failure, and PH is almost reversible if the animal is re-exposed to normal room air (Stenmark et al., 2006). Female favorable adaptation to hypoxia condition with better hemodynamic profile and decrease RV hypertrophy, has also been observed in large animals, as in swine (McMurtry et al., 1973) and chicken (Burton et al., 1968).

In the MCT model, an inflammatory insult producing damage to the PAECs seems to be the primary trigger of PH (Rosenberg and Rabinovitch, 1988). As in the previous hypoxia model, female sex is protective in the rat model of MCT-PH and ovariectomy aggravates PH while exogenous administration of estrogens, in both males and females, improves RV function (Tofovic et al., 2006; Umar et al., 2011). Estradiol metabolites such as 2 ME or 2-hydroxyestradiol are able to prevent and retard the progression of MCT-induced PH in rats by attenuating pulmonary vascular remodeling and RV hypertrophy, and reducing proliferative and inflammatory responses in the lungs (Tofovic et al., 2005; Tofovic et al., 2010). The use of an ERα agonist rescued MCT-PH and protected RV myocardium by restoring RV apelin and bone morphogenetic protein receptor type 2 (BMPR2) (Frump et al., 2021). In the same direction, the administration of an ERβ antagonist abolished the beneficial effect of estrogen whereas the ERβ agonist was as effective as estrogen in rescuing PH (Umar et al., 2011). Also apelin treatment has been shown to significantly attenuate RV hypertrophy and diastolic disfunction in this model (Falcão-Pires et al., 2009).

The hypoxia-sugen model (SuHx) replicates more closely the characteristics of human PAH. In rats, this model presents similar histological lesions as that observed in final stages of the disease in humans (Abe et al., 2010). The vascular endothelial growth factor 2 inhibitor sugen affects the pulmonary vasculature and also interacts with other enzymes like CYP1B1, leading to the production of promitogenic metabolites that contribute to the development of PH (Taraseviciene-stewart et al., 2001). Interestingly, under this model female rats present, despite a more pronounced increase in medial thickness in the small pulmonary arteries, lower inflammatory infiltration of pulmonary arteries and RV fibrosis, and better survival than male rats (Rafikova et al., 2015). This improved RV performance in SuHx females as compared with males, has been shown to be associated with a more favorable antioxidant, anti-inflammatory, antifibrotic and pro-angiogenic profile (Lahm et al., 2016). Moreover, E2 administration has been shown to prevent RV dysfunction and improve pulmonary arterial compliance (Nuedling et al., 2003) through upregulation of matrix-degrading enzymes ADAM15, ADAM17 and osteopontin (Eghbali et al., 2012), and apelin (Frump et al., 2015; Frump et al., 2021). On contrast, in the study by Mair KM et al., the inhibition of ERα had a therapeutic effect on hypoxia-induced PH in female mice and SuHx female rats (Mair et al., 2014) and the use of anastrozole, an inhibitor of the aromatase enzyme, attenuated PH (Mair et al., 2014).

In the bleomycin-induced PH, a model that exhibits some features of interstitial pulmonary fibrosis and PAH, ovariectomy exacerbated the disease and increased mortality in rats, whereas 2 ME tended to reduce mortality and in surviving animals reduced RV systolic pressure and hypertrophy, and attenuated pulmonary inflammation and fibrosis (Tofovic et al., 2009).

In the mouse model of pulmonary artery banding, which is a model of RV pressure overload without concomitant pulmonary vascular disease (nor PH), survival in male mice is improved with reduction of testosterone by castration (Hemnes et al., 2012). Using the same model, very recently, Cheng et al. compared male and female rats with a loss-of-function mutation in ERα finding that female ERα mutants, but not male, developed RV-pulmonary artery uncoupling, RV diastolic dysfunction and fibrosis, thus suggesting that ERα is protective in females (Cheng et al., 2020).

Several mutant and transgenic rodent models of PH have been described. In the model generated by deletion of endothelial nitric oxide synthase, both male and female develop signs of PH during the fetal stage but pathological changes only persist in male mice (Miller et al., 2005). Similar poorer outcomes for males are observed in the lacking vasoactive intestinal peptide model where only males present PH with increased RV hypertrophy and mortality (Said et al., 2007). However, not all the animal models favor female sex. Female mice presenting overexpression of the serotonin transporter SERT, implicated in PASMC proliferation and vasoconstriction (Araujo et al., 2004), exhibit a higher penetrance of PH and its development an severity decreases after ovariectomy (White et al., 2011). In addition, in this model the administration of ERα antagonist attenuates the development of PH in female mice associated with an increase in BMPR2 in the lung (Wright et al., 2015). Similarly, in the PH model induced by S100A4/Mts1 (overexpression of the calcium binding protein) PVR increases and the development of plexiform-like lesions occurs exclusively in female mice (Dempsie et al., 2011).

In summary, animal models reveal that sex significantly modify the expression of experimental PH. However, the results obtained from the different experimental animal models are heterogeneous and occasionally contradictory. Thus, whereas in the classic models of hypoxia, MCT and Su-Hx female sex and estrogens supply are protective, and the same occurs in some transgenic mice models, the opposite happens in other transgenic models such as SERT and S100A4/Mts1. Consequently, the effect of antagonists and agonists of the different ERs varies. Likewise, the effect of sex and estrogens varies at the level of the pulmonary vasculature and cardiac performance. This evidence highlights first, the complexity of PH as an entity and the relevance of evaluating not only pulmonary hemodynamics but also the cardiac function, since the degree of RV adaptation is the main prognostic factor; second, the difficulty understanding the hormonal system and the implication that age, menstrual and reproductive cycle, as well as diet and other extrinsic factors may have; and third, the opposite results that may be obtained from different experimental models, and therefore, the relevance of generating models that reproduce the human physiology as closely as possible, and ideally in large animals.

### Evidence From Human Pulmonary Arterial Hypertension

In patients, estrogens have been typically associated with a higher risk for the development of PAH. There are several studies demonstrating that systemic levels of E2 are higher in patients with PAH as compared to age and body mass-matched controls (Ventetuolo et al., 2016); local estrogen concentration and aromatase enzyme levels in PASMCs from women are higher compared to men (Wright et al., 2015); and absence of hemodynamic differences and RV indices between male and female PAH patients who are older than 45 years-old (Ventetuolo et al., 2011).

Based on this evidence, there are some pilot clinical trials and others ongoing aiming to assess the effect of antiestrogenic drugs in PAH. The third-generation aromatase inhibitor anastrozole reduces E2 and E1 levels. Based on encouraging results in experimental models of PH (Mair et al., 2014; Chen et al., 2017) Kawut et al. randomized 18 patients to anastrozole vs. placebo finding an improvement in the 6-min walking distance (6MWD) test in the anastrozole arm, although no effects were observed in RV function, functional class or health-related quality of life. There are currently a larger ongoing trial (NCT03229499) aiming to evaluate the effects of anastrozole in 6MWD in a larger cohort of patients (*N* = 84). These are undoubtedly very expected results, but we must be cautious since it is unknown whether long-term inhibition of E2 synthesis might have a detrimental effect, mainly at the RV level. In fact, in the pilot randomized clinical trial (Kawut et al., 2017), some patients receiving anastrozole had a worsening of RV function, although globally differences in RV function did not significantly differ as compared with placebo. Another trial (NCT03528902) is evaluating the use of tamoxifen, an antiestrogenic molecule currently used for the treatment of breast cancer, in 24 patients suffering from PAH. The main purpose of the trial is to evaluate the tolerability and impact on functional condition and selected biomarkers of tamoxifen in this population, being the primary outcome the tricuspid annular plane systolic excursion (TAPSE) by echocardiogram, a measure of RV function. Finally, another approach being evaluated is the use of fulvestrant, an ERα inhibitor used in the metastatic breast cancer. It reduces ERα expression, blocks dimerization of this receptor and limits nuclear translocation of transcriptional activating factors (Howell et al., 2000). Recently it has been used for the first time in humans in a small open label proof-of-concept clinical trial in five postmenopausal women with PAH (NCT02911844) (Kawut et al., 2019) showing an improvement in the 6MWD and higher stroke volume without changes in the functional status nor TAPSE or RV systolic pressure. Interestingly a decrease in 16OHE2 was observed.

On the other hand, there is great controversy regarding the effect of hormone replacement therapy (HRT) in PAH. Some observational studies have shown a high prevalence of exposure to HRT in women with PAH suggesting a potential role in the pathogenesis of the disease. Thus, in a questionnaire performed to 88 PAH patients attending a Pulmonary Hypertension Association conference, a high percentage were taking HRT (Lori Sweeney, 2009). Also there is at least one case report in which HRT initiation was associated with PH development in inherited PAH carriers (Morse et al., 1999). On the contrary, in healthy postmenopausal women using HRT, higher levels of E2 were associated with better RV systolic function (Ventetuolo et al., 2011). In a small retrospective study of patients suffering systemic sclerosis (*N* = 61), the use of HRT was associated with a lower incidence of PH (Beretta et al., 2006). Based on this conflicting data, nowadays, international guidelines recommend HRT use only if the patient presents severe menopausal symptoms (Galiè et al., 2015) and, to our knowledge, there are no ongoing trials aiming to evaluate the efficacy of HRT in PAH patients.

The effect of sex, sex hormones, or modifying therapies on PAH cannot be examined without considering the impact they may have on RV, since this is the main prognostic factor. In humans, as occurs in the Su-Hx model, the effect of estrogens can be dimorphic. In this sense, some studies have shown a positive correlation between estrogens levels and RV ejection fraction in women (Ventetuolo et al., 2011). Men present lower RV ejection fraction and higher mean PAP, PVR and right atrial pressure compared to women (Ventetuolo et al., 2011; Shapiro et al., 2012; Ventetuolo et al., 2014) and worse adaptation of the RV to increased pressure overload (Jacobs et al., 2014). These differences may be related to a 5–8% higher mortality rate (Shapiro et al., 2012). Even if testosterone produces a vasodilatory response in isolated pulmonary arteries (Rowell et al., 2009) and higher estrogen/testosterone ratio has been associated to the development of PAH (Wu et al., 2018), it promotes myocardial hypertrophy and in the long-term myocardial fibrosis leading to a maladaptive RV response (Hemnes et al., 2012). All this effects could explain why PAH incidence is lower in men compared to women, but once the disease is stablished RV adaptation and prognosis is worse. A concept called three-tier concept, proposed by Tofovic and Jackson (2020), offers an explanation for the contradictory effects of estrogens observed in experimental animal models and PAH patients. Thereby, estrogens could act as instigators and perpetuators of vascular injury in pulmonary circulation, leading to PAH but also as protectors of RV function, and therefore explaining the greater survival seen in women. The same authors suggest, based on accumulating evidence, that 2 ME acts as a biological antagonist of E2 providing vascular and RV protection.

Besides higher levels of E2, lower levels of DHEA-S have been associated with PAH in men (Ventetuolo et al., 2016) and women (Baird et al., 2018). Declined DHEA levels with age (Kushnir et al., 2010) could partially explain the observed equalization in the ratio men to women of PAH in patients older than 65 years (Hoeper et al., 2013). Apelin expression has been found also reduced in RV homogenates from patients with RV failure (Frump et al., 2021). Based on previous encouraging results from preclinical investigations (Falcão-Pires et al., 2009), a short-term study with the use of apelin in PAH patients was carried out by Brash et al. (NCT01457170) (Brash et al., 2018). Apelin infusion through right heart catheterization reduced PVR and increased cardiac output without changes in mean PAP, pulmonary artery wedge pressure or heart rate. Surprisingly, a post-hoc analysis showed a higher increase in cardiac output and greater reduction in RVP in patients under concomitant treatment with phosphodiesterase-5 inhibitors. A possible action of apelin through the nitric oxide pathway (Salcedo et al., 2007) may be related to a synergic effect with phosphodiesterase-5 inhibitors. No serious adverse events were recorded in this small pilot trial.

Specific subtypes of PAH present similar distribution regarding sex. Mutations in BMPR2 are the most common genetic cause of PAH, occurring in 75–80% of heritable PAH cases but also in 20–25% of idiopathic cases (Machado et al., 2009). PAH related to BMPR2 is inherited in an autosomal dominant manner and it has been reported to present a genetic anticipation trait, that is, disease develops at younger ages in subsequent generations (Loyd et al., 1995). In men, extra regulation by Y chromosome can contribute to a better performance of BMPR2 and lower expression of heritable PAH (Yan et al., 2018). Female patients with connective tissue diseases has been describe to present a higher risk (multiplied by 9) of developing PH than men (Humbert et al., 2006; Chung et al., 2010). In the particular case of systemic sclerosis (SSc) around 12% of patients will develop PAH (Mukerjee et al., 2003), being the incidence higher in women (Mukerjee et al., 2003) but the survival rate lower in males (Chung et al., 2014). However, contrary to other types of PAH, aging seems to be a risk factor for the development of SSc-PAH. Thus, Scorza et al. reported that postmenopausal women suffering from SSc presented a higher risk of developing PAH than the younger ones (Scorza et al., 2002) and HRT could prevent it (Beretta et al., 2006). Single nucleotide polymorphisms of the aromatase enzyme, estrogen receptor 1, and angiopoietin 1 (Roberts et al., 2009) are linked to the development of portopulmonary hypertension. In congenital heart disease, several registries showed a higher prevalence of PH in women, reaching 73.6% of the patients in the Reveal registry (Badesch et al., 2010) and 67.1% in the French registry (Humbert et al., 2006). In Schistosomiasis infection, probably the most common cause of PAH in the world, no gender bias has been observed (Graham et al., 2014). Although the pathobiological mechanisms of the disease are not completely understood, hormonal physiopathology seems not to be involved. Other subtypes of PAH present balanced distribution, with a subtle higher men prevalence in amphetamine-induced (Zamanian et al., 2018) and HIV infection PH (Sitbon et al., 2008). Finally, dysregulations in the immune system (Tamosiuniene et al., 2018) and iron deficiency (118), both problems more prevalent in women, are also associated with the development and progression of PH (Ruiter et al., 2011; Tamosiuniene et al., 2018).

### Sex Differences in Response to Pulmonary Arterial Hypertension Specific Therapies

Part of the estrogen paradox refers to the fact that female patients present better response to PAH treatment and longer survival than men (Shapiro et al., 2012). An extraordinary progress in the discovery on new therapeutic targets for PAH has been made in the last decades. Thereby, besides diuretics, anticoagulation, oxygen, and general measures like physical activity, pregnancy avoidance, psychosocial support and the use of calcium channel blockers in patients with positive vasoreactivity test (Galiè et al., 2015), treatment for PAH has developed on three main pathways: endothelin (endothelin receptors antagonists, ERAs), nitric oxide (phosphodiesterase-5 inhibitors and guanylate cyclase stimulators) and prostacyclin (prostacyclin analogues and prostacyclin receptor agonist).

A pooled analysis of six randomized clinical trials including 1130 PAH patients randomized to ERAs or placebo, showed a significantly greater placebo-adjusted response to ERAs in women in terms of change in 6MWD than men (Gabler et al., 2012). The authors hypothesized that this difference in response could be related to basal disparities in circulating endothelin-1 levels and/or in pulmonary vasoconstriction. No relevant differences in drug pharmacokinetics have been demonstrated (Bruderer et al., 2013). On the contrary, a sub-analysis from The Pulmonary arterial Hypertension and Response to Tadalafil (PHIRST)-I trial (Mathai et al., 2015) showed a better outcome in quality of life and a longer 6MWD in men compared to women. In this case, the authors hypothesized that it could be related to underlying differences in nitric oxide metabolism between men and women. By the use of riociguat, no differences regarding sex was observed in the Pulmonary arterial hyperTENsion sGC-stimulator Trial (PATENT)-1 (Ghofrani et al., 2013). Finally, in patients requiring the use of epoprostenol, male presented a higher hospitalization time and lower survival (Frantz et al., 2015). Interestingly, Jacobs et al. (2014) reported that 1 year after initiation of PAH-specific therapy, although male and female patients showed a similar reduction in PVR, RV ejection fraction improved in female patients whereas it deteriorated in male patients, thereby suggesting that differences in RV ejection fraction response after initiation of medical therapy could explained a significant portion of the worse survival seen in men. The small sample size limited the ability to perform sex-specific analysis for the different therapeutic groups. The identification of treatment-response heterogeneity can help to take individual treatment decisions and highlights the relevance of stratifying by sex in future randomized clinical trials.

## Sex Differences in Other Pulmonary Hypertension Groups

Evidence regarding sex differences in other groups of PH is scarce despite significantly more prevalent than PAH.

### Pulmonary Hypertension Group 2

PH secondary to left heart disease or PH group 2 is the most common type of PH. Its prevalence can reach 73% in patients suffering HF with reduced ejection fraction (Miller et al., 2013), and 83% of those with preserved ejection fraction (Shah et al., 2014). Even if the latter is a disease with a clear female predominance (Dewan et al., 2019), a more severe PH and RV dysfunction has been observed in male suffering this disease (Melenovsky et al., 2014; Duca et al., 2018). Consequently, cardiac related mortality has been strongly associated with male sex in this scenario but not all-cause mortality, which is similar between sexes (Duca et al., 2018) or even greater in females (Sotomi et al., 2021). Once again, worse adaptation of the RV to pressure and/or volume overload seems to be the key to explain worse outcomes in males. In this sense, data from the Veterans Affairs Clinical Assessment Reporting and Tracking (CART) study described a better RV adaptation and survival in women compared to men in the group of patients with PH group 2 (Ventetuolo et al., 2017). In the case of HF with reduced ejection fraction, women also showed less RV dysfunction despite similar hemodynamic parameters and left ventricular ejection fraction (Martínez-Sellés et al., 2006). Better RV compliance and ability of women to manage volume overload better, particularly in women with previous pregnancies, has been speculated to contribute (Martínez-Sellés et al., 2006).

### Pulmonary Hypertension Group 3

Estimated prevalence of PH secondary to chronic lung disease varies from 50 to 70% of patients with moderate to severe chronic obstructive pulmonary disease (COPD) and emphysema (Scharf et al., 2002; Chaouat et al., 2008). COPD prevalence is higher in men, but there may be a bias due to differences in smoking habits in the past (Yin et al., 2017). PH in COPD develops in patients with severe airflow limitation due to chronic hypoxemia and it confers a worse prognosis (Cuttica et al., 2010). In idiopathic pulmonary fibrosis, the incidence of PH increases with the severity of the disease, from 8 to 15% at diagnosis to 30–50% in advanced stages, and men are more prone to be affected than women (Kimura et al., 2013). Again, in this group of PH, male sex is a risk factor for RV dysfunction, even after adjusting by RV pressure afterload (Prins et al., 2019).

### Pulmonary Hypertension Group 4

In an observational study aimed to estimate the incidence of risk factors of CTEPH in a cohort with first venous thromboembolism (*N* = 23,329 patients), female sex was significantly associated with the development of the disease (Martinez et al., 2018). However, studies carried out in Spanish and Chinese cohorts have showed a better survival in women as compared with their male counterparts (Escribano-Subias et al., 2012; Chen et al., 2018). This improved survival could be related to better cardiac performance, with lower atrial pressure and higher cardiac index to similar PVR compared to men (Shigeta et al., 2008). However, during follow-up female have been shown to present greater hemodynamic deterioration than men (Yang et al., 2020). This fact, however, could be related to different factors. Women are less likely to receive a surgical treatment or to be classified as operable than men, especially at low-volume centers for this pathology (Barco et al., 2020), although these differences could be also related to a longer diagnostic delay and a more advanced stage of the disease at the time of diagnosis (Barco et al., 2020).

## Conclusion

PH includes a variety of diseases currently classified according to its etiology and hemodynamic profile. Sex differences are observed in practically all groups of PH. The estrogen paradox in PAH refers to the fact that women present a higher risk of disease development but once affected, they present a better response to treatment and longer survival as compared to men. Evidence regarding estrogens effects in PH animal models shows contradictory results, both protective and deleterious. The dimorphic effect of estrogens over the pulmonary vasculature and RV performance may explain the paradox, particularly the impact on RV function of sex, hormones, and response to specific treatment seems to be the key to understand the better prognosis of PH in women compared to men. Finally, even if the main reason for this sex bias may correspond to sex hormones, other factors can play an important role in the disease development, such as comorbidities burden or differences in the clinical and pharmacological management. Further studies, both in PAH and other PH groups should be encouraged to better understand the influence of sex in this disease.
